# Serial Changes in Blood-Cell-Count-Derived and CRP-Derived Inflammatory Indices of COVID-19 Patients

**DOI:** 10.3390/diagnostics13040746

**Published:** 2023-02-16

**Authors:** Maryam B. Khadzhieva, Alesya S. Gracheva, Olesya B. Belopolskaya, Yulia V. Chursinova, Ivan V. Redkin, Mikhail V. Pisarev, Artem N. Kuzovlev

**Affiliations:** 1 Federal Research and Clinical Center of Intensive Care Medicine and Rehabilitology, 107031 Moscow, Russia; 2 Dmitry Rogachev National Research Center of Pediatric Hematology, Oncology and Immunology, 117997 Moscow, Russia; 3 Vavilov Institute of General Genetics, Russian Academy of Sciences, 119991 Moscow, Russia; 4 Resource Center “Bio-Bank Center”, Research Park of St. Petersburg State University, 198504 St. Petersburg, Russia; 5 Federal Scientific and Clinical Center of Medical Rehabilitation and Balneology of the Federal Medical and Biological Agency of Russia, 127410 Moscow, Russia

**Keywords:** COVID-19, mortality, aggregate index of systemic inflammation (AISI), C-reactive protein to lymphocytes ratio (CLR), lymphocyte to monocyte ratio (LMR), multi-inflammatory index (MII), neutrophil to lymphocyte ratio (NLR), platelet to lymphocyte ratio (PLR), systemic inflammatory index (SII), systemic inflammation response index (SIRI)

## Abstract

The aim of the study was to investigate the serial changes in inflammatory indices derived from blood cell counts and C-reactive protein (CRP) levels in COVID-19 patients with good and poor outcomes. We retrospectively analyzed the serial changes in the inflammatory indices in 169 COVID-19 patients. Comparative analyses were performed on the first and last days of a hospital stay or death and serially from day 1 to day 30 from the symptom onset. On admission, non-survivors had higher CRP to lymphocytes ratio (CLR) and multi-inflammatory index (MII) values than survivors, while at the time of discharge/death, the largest differences were found for the neutrophil to lymphocyte ratio (NLR), systemic inflammation response index (SIRI), and MII. A significant decrease in NLR, CLR, and MII by the time of discharge was documented in the survivors, and a significant increase in NLR was documented in the non-survivors. The NLR was the only one that remained significant from days 7–30 of disease in intergroup comparisons. The correlation between the indices and the outcome was observed starting from days 13–15. The changes in the index values over time proved to be more helpful in predicting COVID-19 outcomes than those measured on admission. The values of the inflammatory indices could reliably predict the outcome no earlier than days 13–15 of the disease.

## 1. Introduction

Systemic inflammation in the novel coronavirus infection COVID-19 results in acute respiratory distress syndrome (ARDS), disseminated intravascular coagulation, and multiple organ failure, associated with severe illness and adverse outcomes [1,2,3]. The common blood count is a simple, inexpensive, and rapid tool for the evaluation of inflammation in the early diagnosis and prognosis of diseases [4,5,6]. The dynamic nature of inflammation in COVID-19 is crucial and relates directly to physiological parameters [7]. In severe COVID-19, changes in several parameters of the common blood count have been reported, such as elevated leukocyte and neutrophil counts, increased RDW, and persistent decreases in lymphocytes and platelets [8,9,10,11,12]. Neutrophils are the most abundant and mobile immune cells in human blood, constituting approximately 50–70% of the total white blood cell count. Neutrophils represent the first line of defense against pathogens and are therefore an important component of the innate immune response. However, as a result of poorly controlled activation, they can also mediate tissue damage in multiple diseases, often by increasing inflammation and tissue damage [13]. In viral infections, the immune response is orchestrated largely by lymphocytes, while the total count of lymphocytes and their subpopulations varies depending on the type of virus. Thus, lymphopenia in severe COVID-19 is mainly due to a significant decrease in T-lymphocytes [14]. Platelets are critical to hemostasis and thrombosis and participate in other physiological and pathological processes such as inflammation, infection, cancer metastasis, and the maintenance of vascular integrity during inflammation [15]. C-reactive protein (CRP) is a nonspecific acute phase protein and a sensitive biomarker of acute infection, inflammation, and tissue damage [16]. In patients with severe COVID-19, there is a remarkable increase in serum CRP, indicating an enhanced systemic inflammatory response [17,18]. The ratios of different components of complete blood count and CRP have also been studied as inflammatory markers for the diagnosis, severity assessment, and prognosis of inflammatory diseases [19,20,21].

Since COVID-19 is characterized by a wide range of clinical manifestations and variability of progression, the study of the serial changes in potential predictors of severe disease and lethal outcome is important for optimal treatment depending on the stage of the disease [22]. The use of prognostic scales developed based on the correlation of the serial changes of clinical and laboratory parameters with morphological data can help in the prompt assessment of possible clinical disease variants and the identification of patients at high risk of poor outcomes [23]. In our study of patients with COVID-19 with good and poor outcomes, a serial assessment of such blood-cell-count-related and CRP-related inflammatory markers as lymphocyte to monocyte ratio (LMR), neutrophil to lymphocyte ratio (NLR), platelet to lymphocyte ratio (PLR), C-reactive protein to lymphocytes ratio (CLR), aggregate index of systemic inflammation (AISI), multi-inflammatory index (MII), systemic inflammatory index (SII), and systemic inflammation response index (SIRI) was performed.

## 2. Materials and Methods

### 2.1. Patients

The retrospective study included 169 patients older than 18 years hospitalized at the M.F. Vladimirsky Moscow Regional Clinical Hospital and the Voronovskoye Infectious Disease Hospital with the diagnosis of COVID-19 from 26 April to 1 December 2020. The diagnosis was confirmed by laboratory testing using RT-PCR of nasopharyngeal and oropharyngeal smears in all patients. The inclusion criteria were patients of both sexes older than 18 years; consent to participate in the study and the completion of an appropriate informed consent form; the absence of pregnancy; and the absence of severe medical, immunological, and surgical comorbidities during the study. The exclusion criteria were terminal incurable conditions, pregnancy, and refusal to participate in the study. The patients were divided into two groups depending on the outcome. The demographic, clinical, and laboratory data were obtained from the medical record system.

### 2.2. Statistical Analysis

The inflammatory indices were calculated as follows:

Aggregate index of systemic inflammation (AISI) = Neutrophil count × monocyte count × platelet count/lymphocyte count
C-reactive protein to lymphocytes ratio (CLR) = C-reactive protein (CRP)/lymphocyte count
Lymphocyte to monocyte ratio (LMR) = Lymphocyte count/monocyte count
Neutrophil to lymphocyte ratio (NLR) = Neutrophil count/lymphocyte count
Multi-inflammatory index (MII) = NLR × CRP
Platelet to lymphocyte ratio (PLR) = Platelet count/lymphocyte count
Systemic inflammatory index (SII) = Neutrophil count × PLR
Systemic inflammation response index (SIRI) = Neutrophil count × monocyte count/lymphocyte count

These inflammatory indices were calculated serially from admission to the hospital or ICU with a diagnosis of COVID-19 until the discharge or in-hospital death of the patient. A CT (computed tomography of the lungs) severity score was assigned depending on the extent of the lung lesions (semiquantitatively): 0 corresponded to no lesion (0%), 1 corresponded to minimal (1–25%), 2 corresponded to mild (26–50%), 3 corresponded to moderate (51–75%), and 4 corresponded to severe (76–100%) lesions.

The statistical analysis and data visualization were performed using Statsoft Statistica version 13 and R statistical software version 4.0.3. The quantitative nonparametric variables were presented as the median and 25th and 75th percentiles and as values and percentages for the categorical data. The nonparametric Mann–Whitney U-criterion was used for intergroup analysis, and the Wilcoxon criterion was used to analyze the serial changes of the parameters. Fisher’s exact two-sided F-criterion was used to analyze the binary data, and the correlation analysis was performed by calculating the Spearman correlation coefficient. The significance level at which the null hypothesis of no difference between the study groups was rejected was taken to be 0.05. Logistic regression analysis and ROC analysis were used to estimate the examined inflammation indices as predictors of fatal outcome. The optimal cutoff point was determined using Youden’s index. The correction for multiple comparisons was performed using the Benjamini–Hochberg method (FDR, false discovery rate). We established the test power in the range of 89.0% (CLR) to 99.8% (NLR) for indices in the intergroup comparisons on the last day of the hospital stay or death, whereas the statistical power was 17.3% for MII and 19.1% for CLR on the first day of the hospital stay.

## 3. Results

### 3.1. Patient Characteristics

The total sample of 169 patients older than 18 years with confirmed COVID-19 was retrospectively divided into two groups based on the outcomes: 138 survivors and 31 non-survivors with the median ages (25th and 75th percentiles) of 57.00 (46.00 to 65.00) and 62.00 (59.00 to 70.00) years, respectively (*p* = 0.0154). Of the total sample, 53.85% were male; there were no intergroup differences by sex. Table 1 presents the main characteristics of the patients included in the study. The groups did not differ significantly in the frequency of comorbidities, as well as in the number of days from the onset of symptoms to admission, length of stay in the hospital or ICU, and the severity of the lung lesions on the CT scan. The period of hospitalization ranged from 3 to 62 days, with the median (25th and 75th percentiles) of 15 (11 to 22) days. The oxygen saturation (SpO2) on admission was lower in the non-survivors group than in the survivors (90% [87–95%] versus 94% [91–96%], *p* = 0.0278). Acute respiratory distress syndrome (ARDS) developed more frequently in the non-survivors group (*p* = 1.00 × 10^−5^). Differences were also found in the absolute neutrophil counts (*p* = 0.0361) and CRP levels (*p* = 0.0446) on the day of admission.

### 3.2. Serial Changes in Inflammatory Indices in COVID-19 Patients with Different Outcomes

**Figure 1:**
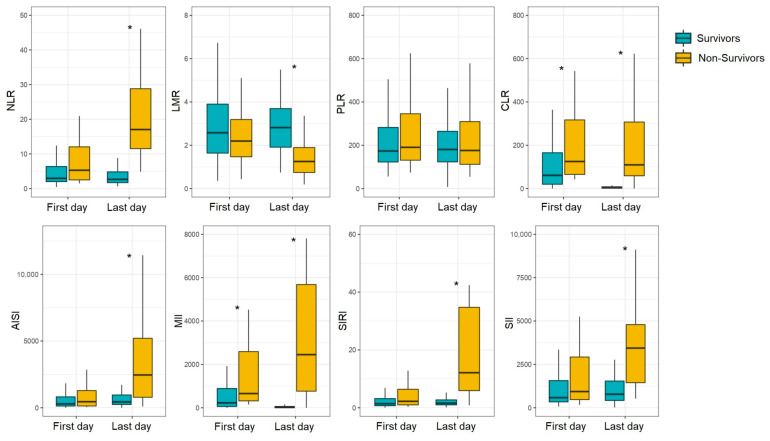
Blood-cell-count- and CRP-derived inflammatory indices in COVID-19 patients on the first and last days of hospital stay. * FDR adj. *p*-value < 0.05.

We performed an intergroup comparison of the NLR, LMR, PLR, CLR, AISI, MII, SIRI, and SII values at the time of admission with a COVID-19 diagnosis and at the day of discharge or hospital death (Figure 1, Supplementary Table S1). On admission to the hospital/ICU, the NLR, CLR, and MII values were significantly higher in non-survivors than in survivors (NLR, 5.28 vs. 2.95, *p* = 0.0441; CLR, 125.00 vs. 61.01, *p* = 0.0273; MII, 657.02 vs. 227.43, *p* = 0.0085). After adjusting for multiple comparisons, differences persisted for CLR (FDR adj. *p*-value = 0.0485) and MII (FDR adj. *p*-value = 0.0169). At the time of the patient’s discharge or hospital death, the differences were recorded for all indices studied except PLR. The largest differences were found for NLR (2.66 in survivors vs. 17.04 in non-survivors, FDR adj. *p*-value = 4.34 × 10^−20^), SIRI (1.60 in survivors vs. 12.13 in non-survivors, FDR adj. *p*-value = 6.70 × 10^−14^), and MII (16.91 in survivors vs. 2450.44 in non-survivors, FDR adj. *p*-value = 3.57 × 10^−10^).

When comparing the inflammatory indices on the first and last days of the hospital stay, a decrease in NLR (*p* = 0.0242) was observed in the surviving patients, while a significant increase in this parameter (*p* = 3.41 × 10^−5^) was characteristic of the non-surviving patients. There was also a significant decrease in CLR (*p* = 4.69 × 10^−10^) and MII (*p* = 7.66 × 10^−10^) by the time of discharge in the survivors group. For patients with poor outcomes, there was a decrease in LMR (*p* = 0.0060) and an increase in SIRI (*p* = 3.11 × 10^−5^) and SII (*p* = 0.0041). An increase in AISI in both groups (*p* = 0.0203 in survivors; *p* = 0.0005 in non-survivors) was seen (Supplementary Table S2). After adjusting for multiple comparisons, the differences were still significant.

The correlation analysis of the inflammatory indices with the severity of the lung lesions on the CT scan on admission revealed a Spearman correlation coefficient of at least 0.5 only for MII (for both survivors and non-survivors) and CLR (for survivors), due primarily to the contribution of the CRP levels (Table 2).

**Figure 2:**
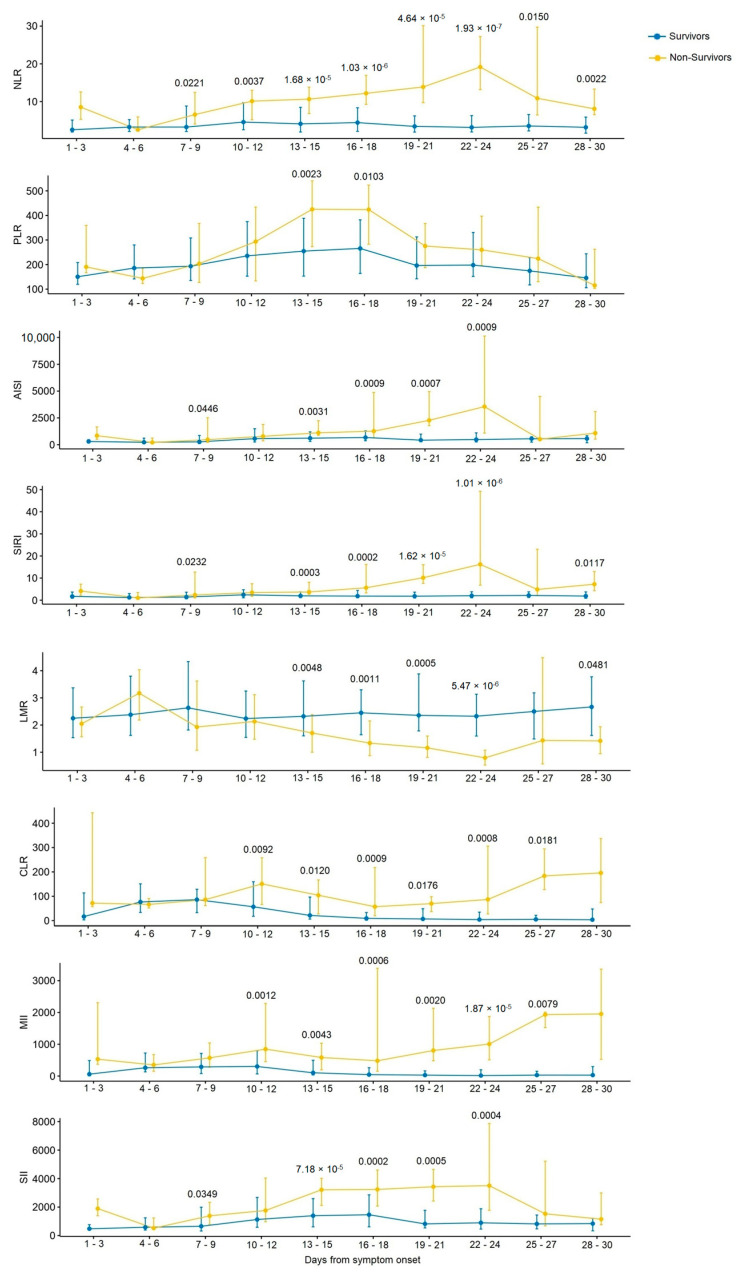
Serial changes in blood-cell-count- and CRP-derived inflammatory indices in COVID-19 patients. *p*-values less than 0.05 are indicated.

We calculated the COVID-19 inflammatory indices during 30 days from the onset of the symptoms to trace their evolution (Figure 2, Supplementary Table S3), in three-day increments. The earliest differences between patients based on the disease outcomes were for NLR, AISI, SIRI, and SII (on days 7 to 9 of disease). The NLR levels in the patients with good outcomes remained almost unchanged over the course of the disease, whereas an increase in this parameter was noted in non-survivors. For all inflammatory indices except PLR, after adjusting for multiple comparisons, intergroup differences were found from day 13 to 24 of the disease. For both survivors and non-survivors, there was a gradual increase in PLR until days 16–18 and a decrease from day 19 to 30 of the disease, with differences between the patient groups first detected on days 13–18. The NLR was the only parameter remaining significant from days 7 to 30 of disease after adjusting for multiple comparisons (Supplementary Table S3).

### 3.3. Inflammatory Indices as Potential Predictors of In-Hospital Mortality in COVID-19 Patients

**Figure 3:**
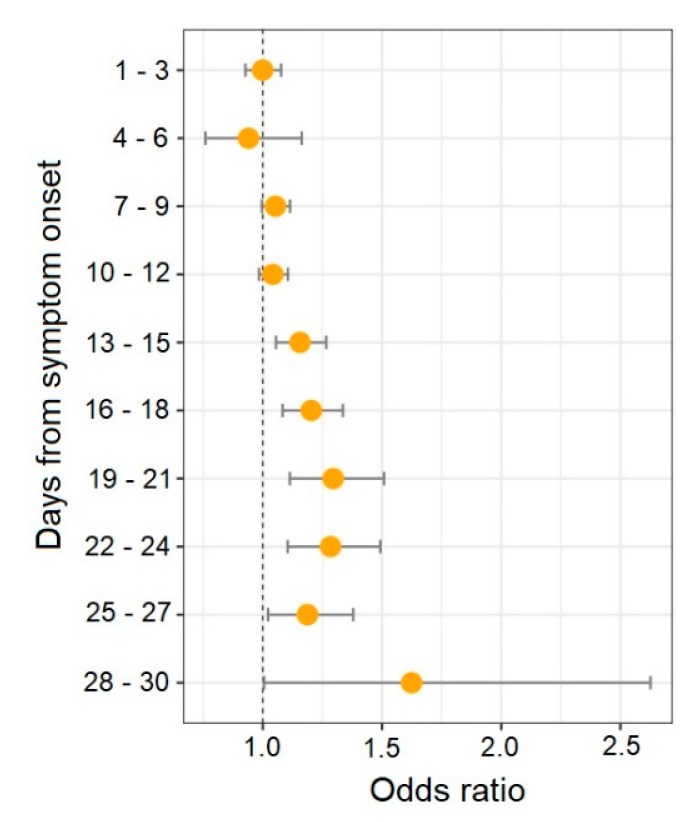
Association between serial changes of NLR and mortality in COVID-19 patients (adjusted odds ratio and 95% confidence interval).

The evaluation of inflammatory indices as potential predictors of lethal outcomes was performed for the indices that differed significantly between the patient groups. According to the results of the age- and sex-adjusted logistic regression analysis of the indices across time, the best predictors of mortality were NLR on days 19 to 21 of illness (OR: 1.296; 95% CI: 1.113–1.508; *p* = 0.001), days 22 to 24 (OR: 1.284; 95% CI: 1.104–1.493; *p* = 0.001), and days 28 to 30 (OR: 1.624; 95% CI: 1.005–2.626; *p* = 0.048) and SIRI on days 19 to 21 of illness (OR: 1.386; 95% CI: 1.164–1.651; *p* = 0.0002). Meanwhile, a protective effect of LMR on days 13–15 (OR: 0.573; 95% CI: 0.356–0.923; *p* = 0.022) and days 22–24 (OR: 0.101; 95% CI: 0.025–0.413; *p* = 0.001) was found (Supplementary Table S3). The bulk of the associations were recorded from days 13 to 18. The NLR is the only index for which a correlation with mortality was reported from day 13 to 30 from the symptom onset (Figure 3).

The results of the ROC analysis, predictive accuracy, and optimal cutoff points are presented in Supplementary Table S4. A high diagnostic performance (AUC ≥ 0.8, sensitivity and specificity ≥ 0.7) in at least one group of days was demonstrated for NLR, SIRI, SII, MII, AISI, and CLR. Until days 16–18 after the symptom onset, none of the inflammatory indices had an AUC ≥ 0.8. The highest AUC (≥0.9) was recorded on days 22 to 24 for NLR (0.930), MII (0.909), and SIRI (0.912) and on days 25 to 27 for MII (0.950) and CLR (0.917).

## 4. Discussion

We examined the serial changes in such inflammatory indices as LMR, NLR, PLR, CLR, AISI, MII, SII, and SIRI in COVID-19 patients with good and poor outcomes. The CLR and MII values were higher in non-survivors than in survivors on admission. These parameters also correlated with the CT score in the overall sample. The CLR index is calculated as the ratio of CRP to the absolute number of lymphocytes. This parameter has been considered as a predictor of hospital death in aortic dissection [24] and adverse outcomes of cancer [25,26,27] and as a discrimination tool between acute and perforated appendicitis [28]. Several studies have shown that high CLR values are characteristic of patients with severe COVID-19 [29,30]. The MII index is the product of NLR and CRP, which have been considered biomarkers of lethal outcome in COVID-19. This index was proposed in 2020 by A. Casadei Gardini et al. as a predictor of mortality in patients with metastatic colorectal cancer [31]. In the context of the development and severity of COVID-19, MII demonstrated the best performance for predicting mortality among all inflammatory markers studied in the study with no differences in PLR and SII found between survivors and non-survivors [32]. The correlation of CLR and MII with the CT score on admission is probably due to the CRP levels. The association of the CRP levels with the lung lesion extent has been shown both in our study and in the studies of our colleagues [33,34,35]. At the time of discharge/death, the largest differences between survivors and non-survivors were found for NLR, SIRI, and MII. On the last day of hospitalization, compared with values on admission, there was an increase in NLR, SIRI, and SII and a decrease in LMR among the non-survivors. A drop in NLR, CLR, and MII down to normal values at the time of discharge was recorded in the survivor group. These findings are consistent with a study reporting a marked increase in NLR and CLR among patients admitted to the ICU and those who died, compared with patients with moderate severity and healthy controls, and there was a decrease in LMR on the last day of the hospital stay in non-survivors. By the time of recovery, NLR and CLR returned to near normal values, whereas in non-survivors a persistent increase in NLR and CLR was observed until the last day of the ICU stay [30]. B. Cheng et al. also showed that NLR and CRP are good predictors of COVID-19 progression to critical illness and death [36].

On days 7–9 of the disease, differences between survivors and non-survivors were found for NLR, AISI, SIRI, and SII. From days 13 to 24, differences were observed for all inflammatory indices except PLR. Patients with a fatal outcome showed a significant increase in NLR during the hospital stay. This index was the only one that remained significant from day 7 to day 30 of illness in intergroup comparisons. The NLR on days 19 to 30 of illness and the SIRI on days 19 to 21 of illness were the best predictors of a fatal outcome. Our results agree with studies demonstrating the relationship between inflammatory indices and the prognosis of COVID-19. A study of hematologic parameters in patients with COVID-19 in South India revealed an increase in the NLR and PLR values with disease progression, whereas the NLR value of 40.95 and PLR value of 400 were found to be cutoffs for the lethal outcome [37]. Serial changes in D-dimer and NLR proved to be more valuable in predicting COVID-19 than the values of these parameters assessed on admission [38]. In examining the prognostic role of NLR, dNLR, MLR, PLR SIRI, and SII values on the day of admission in COVID-19 outcomes, binary logistic regression revealed elevated NLR, dNLR, and MLR as independent factors of poor clinical outcomes for COVID-19 [39]. The in-hospital mortality in patients with COVID-19 was predicted by SII greater than 1835 on admission [21]. The NLR, PLR, and CLR were higher in COVID-19 patients with pneumonia compared with those without pneumonia, while CLR was more effective than NLR and PLR in discriminating between COVID-19 patients with and without pneumonia [29]. The NLR is an independent biomarker indicating a poor clinical outcome in COVID-19 [40,41]. The meta-regression analysis showed that the association between NLR values on admission and the disease severity in patients with COVID-19 was not affected by age, sex, cardiovascular disease, diabetes mellitus, or hypertension [42]. This index provides additional information about the prolonged inflammatory response in patients with COVID-19, particularly in those with a poor prognosis. Meta-analyses have shown the high prognostic power of NLR for assessing the severity risk of fatal outcomes in COVID-19 [43,44,45], but there is no consensus on the optimal NLR cutoff for elevated levels, especially in patients with COVID-19.

## 5. Conclusions

Our study has several limitations, including a retrospective design and a relatively small sample size. Therefore, these findings warrant confirmation in other populations, particularly by multicenter studies. We studied the serial changes in the values of blood-cell-count-related and CRP-related inflammatory indices such as LMR, NLR, PLR, CLR, AISI, MII, SII, and SIRI. The changes in these indices over time proved to be more valuable in predicting COVID-19 outcomes than their values on admission. The inflammatory indices were not helpful in predicting the disease outcome until at least 13–15 days after the onset of symptoms. The NLR is a potential marker of mortality in COVID-19, providing a simple and rapid tool that can be useful for the management and risk stratification of COVID-19 patients.

## Figures and Tables

**Table 1:** Baseline characteristics of hospitalized patients with COVID-19.

Parameter	Total (*n* = 169)	Survivors (*n* = 138)	Non-Survivors (*n* = 31)	*p*-Value
Age, years	59.00 (48.00–67.00)	57.00 (46.00–65.00)	62.00 (59.00–70.00)	**0.0154**
Male	91/169 (53.85%)	73/138 (52.52%)	18/31 (58.06%)	0.6917
Hypertension	106/169 (62.72%)	82/138 (59.42%)	24/31 (77.42%)	0.0672
Diabetes mellitus	40/169 (23.67%)	30/138 (21.74%)	10/31 (32.26%)	0.2439
Coronary heart disease	42/169 (24.85%)	31/138 (22.46%)	11/31 (35.48%)	0.1666
Obesity	40/169 (23.67%)	30/138 (21.74%)	10/31 (32.26%)	0.2439
Interval between disease onset and admission, days	8 (6–11)	8 (6–11)	7 (5–9)	0.3242
Length of stay in hospital/ICU, days	15 (11–22)	15 (11–21)	13 (9–24)	0.4904
SpO2, %	94 (89–96)	94 (91–96)	90 (87–95)	**0.0278**
Missing, *n* (%)	7/169 (4.14%)	6/138 (4.35%)	1/31 (3.23%)	
CT:				0.0985
0–1	54/169 (31.95%)	45/138 (32.61%)	9/31 (29.03%)	
2	59/169 (34.91%)	51/138 (36.96%)	8/31 (25.81%)	
3	37/169 (21.89%)	29/138 (21.01%)	8/31 (25.81%)	
4	19/169 (11.24%)	13/138 (9.42%)	6/31 (19.35%)	
ARDS	46/169 (27.22%)	20/138 (14.49%)	26/31 (83.87%)	**1.00 × 10^−5^**
WBC (×10^9^/L)	5.99 (4.12–8.01)	5.92 (4.00–8.00)	6.70 (4.60–11.30)	0.2409
Monocytes (×10^9^/L)	0.41 (0.28–0.63)	0.42 (0.29–0.62)	0.41 (0.22–0.66)	0.6778
Lymphocytes (×10^9^/L)	1.11 (0.77–1.46)	1.15 (0.81–1.49)	1.03 (0.51–1.36)	0.1723
Neutrophils (×10^9^/L)	3.91 (2.45–6.31)	3.74 (2.36–6.08)	4.61 (3.17–8.51)	**0.0361**
Platelets (×10^9^/L)	205.00 (168.50–249.00)	205.00 (172.00–252.00)	204.00 (156.00–237.00)	0.3636
CRP (mg/L)	81.20 (32.92–156.60)	69.95 (28.71–153.40)	128.20 (62.10–233.50)	**0.0446**
Missing, *n* (%)	60/169 (35.50%)	44/138 (31.88%)	16/31 (51.61%)	

The data are presented as median and interquartile range (25th–75th percentiles) or *n* of N (%), where N is the total number of patients with available data. *p*-values comparing survivors and non-survivors were obtained using the two-sided Fisher’s exact test or Mann–Whitney U test. Significant results are in bold. ARDS, acute respiratory distress syndrome; CRP, C-reactive protein; CT, computed tomography of the lungs; SpO2, peripheral oxygen saturation; WBC, white blood cell.

**Table 2:** Correlations of CRP and CRP-derived inflammatory indices with CT score on the day of admission.

Correlation Pairs	Total		Survivors		Non-Survivors	
	*ρ*	*p*-Value	*ρ*	*p*-Value	*ρ*	*p*-Value
CRP and CT score	0.6287	2.49 × 10^−13^	0.6317	8.62 × 10^−12^	0.4619	0.0831
MII and CT score	0.6130	3.62 × 10^−12^	0.5956	5.96 × 10^−10^	0.6622	0.0072
CLR and CT score	0.5927	2.71 × 10^−11^	0.5968	5.36 × 10^−10^	0.4563	0.0873

## Data Availability

The data sets used and/or analyzed during the present study are available from the corresponding author upon request.

## Supplementary Materials

The following supporting information can be downloaded at: https://www.mdpi.com/article/10.3390/diagnostics13040746/s1: Table S1: Blood-cell-count- and CRP-derived inflammatory indices in COVID-19 patients on the first and last days of hospital stay (intergroup comparative analysis); Table S2: Blood-cell-count- and CRP-derived inflammatory indices in COVID-19 patients on the first and last days of hospital stay (intra-group comparative analysis); Table S3: Serial changes in blood-cell-count- and CRP-derived inflammatory indices in COVID-19 patients from day 1 to day 30 from the symptom onset; Table S4: Summary statistics of the results of ROC analysis.
